# Operationalizing frailty among older residents of assisted living facilities

**DOI:** 10.1186/1471-2318-11-23

**Published:** 2011-05-13

**Authors:** Elizabeth A Freiheit, David B Hogan, Laurel A Strain, Heidi N Schmaltz, Scott B Patten, Misha Eliasziw, Colleen J Maxwell

**Affiliations:** 1 Department of Community Health Sciences, University of Calgary, 3rdFloor TRW, 3280 Hospital Drive NW, Calgary, Alberta, Canada; 2 Department of Medicine, University of Calgary, HSC 3330 Hospital Drive NW, Calgary, Alberta, Canada; 3 Department of Sociology, University of Alberta, 5-21 HM Tory Building, Edmonton, Alberta, Canada

## Abstract

**Background:**

Frailty in later life is viewed as a state of heightened vulnerability to poor outcomes. The utility of frailty as a measure of vulnerability in the assisted living (AL) population remains unexplored. We examined the feasibility and predictive accuracy of two different interpretations of the Cardiovascular Health Study (CHS) frailty criteria in a population-based sample of AL residents.

**Methods:**

CHS frailty criteria were operationalized using two different approaches in 928 AL residents from the Alberta Continuing Care Epidemiological Studies (ACCES). Risks of one-year mortality and hospitalization were estimated for those categorized as frail or pre-frail (compared with non-frail). The prognostic significance of individual criteria was explored, and the area under the ROC curve (AUC) was calculated for select models to assess the utility of frailty in predicting one-year outcomes.

**Results:**

Regarding feasibility, complete CHS criteria could not be assessed for 40% of the initial 1,067 residents. Consideration of supplementary items for select criteria reduced this to 12%. Using absolute (CHS-specified) cut-points, 48% of residents were categorized as frail and were at greater risk for death (adjusted risk ratio [RR] 1.75, 95% CI 1.08-2.83) and hospitalization (adjusted RR 1.54, 95% CI 1.20-1.96). Pre-frail residents defined by absolute cut-points (48.6%) showed no increased risk for mortality or hospitalization compared with non-frail residents. Using relative cut-points (derived from AL sample), 19% were defined as frail and 55% as pre-frail and the associated risks for mortality and hospitalization varied by sex. Frail (but not pre-frail) women were more likely to die (RR 1.58 95% CI 1.02-2.44) and be hospitalized (RR 1.53 95% CI 1.25-1.87). Frail and pre-frail men showed an increased mortality risk (RR 3.21 95% CI 1.71-6.00 and RR 2.61 95% CI 1.40-4.85, respectively) while only pre-frail men had an increased risk of hospitalization (RR 1.58 95% CI 1.15-2.17). Although incorporating either frailty measure improved the performance of predictive models, the best AUCs were 0.702 for mortality and 0.633 for hospitalization.

**Conclusions:**

Application of the CHS criteria for frailty was problematic and only marginally improved the prediction of select adverse outcomes in AL residents. Development and validation of alternative approaches for detecting frailty in this population, including consideration of female/male differences, is warranted.

## Background

Residents of assisted living (AL) facilities represent a unique population in relation to frailty research. AL is an increasingly important residential care option for older adults in North America [1,2]. AL residents require select health and personal services within a secure residential environment but not the continuous monitoring and more intensive professional care found within nursing homes [2]. There is general agreement that the core feature of frailty is an increased vulnerability to stressors [3] and that such vulnerability is likely to be present to varying degrees within a given population [4]. While AL residents would be expected to exhibit relatively higher levels of vulnerability than similarly aged individuals in the community, within this heterogeneous population varying degrees of frailty will be present. Being able to identify relatively vulnerable AL residents at higher risk of adverse health outcomes, if coupled with effective interventions, would offer opportunities to maximize their independence and quality of life.

Although there is no consensus on how best to identify frailty in an older person, criteria proposed by Fried and colleagues using data from the Cardiovascular Health Study (CHS) have attracted the most interest [3,5,6]. Developed on a cohort of community-dwelling seniors, frailty was defined as the presence of three or more of five criteria (slow gait, muscle weakness as determined by grip strength, low physical activity, unintentional weight loss and self-reported exhaustion) [5]. This index has been linked to physiological alterations believed to underlie the multisystem impairment and decline in homeostatic reserve and resiliency characteristic of the clinical syndrome of frailty [3,7]. Those categorized as frail showed a greater risk of subsequent functional decline, falls, hospitalizations and death [5]. Although argued to provide an objective and easily measured screen for frailty, criticisms of the CHS definition include its restriction to physical characteristics and the exclusion of persons with dementia or on anti-depressants from the sample used to develop the measure [3,8,9]. The AL population is characterized by a relatively high prevalence of dementia and depression [10,11], raising questions about the feasibility and prognostic value of the CHS criteria in this setting.

This is the first paper to examine the operationalization, utility and predictive accuracy of the CHS frailty criteria in an AL population. We compared two different interpretations of these criteria. One used CHS-specified cut-points (*absolute cut-points*) for determining select criteria while the second was norm-referenced and identified the poorest performers for that criterion within the AL population (*relative cut-points*). We hypothesized that the absolute cut-points, developed in a community-based cohort, would be too inclusive and would limit the ability of frailty to discriminate AL residents at higher risk for adverse outcomes. Specific objectives were to: i) determine the feasibility of assessing frailty in AL residents using the CHS criteria; ii) estimate the prevalence of frailty based on CHS criteria and determine associated risks for all-cause mortality and hospitalizations in AL residents; iii) compare the predictive accuracy of the criteria using differing interpretations; and, iv) assess how well models with and without frailty predict mortality and hospitalization.

## Methods

### Study design

This was a sub-study of the Alberta Continuing Care Epidemiological Studies (ACCES) cohort, a provincial study of the health and quality of care issues in AL and nursing home facilities. The ACCES-AL cohort included residents of designated (publicly-funded) assisted living and supportive housing facilities (DAL) in five former health regions (two urban, three rural) in the province of Alberta, Canada.

These facilities offer a wide array of services in diverse settings and often provide a combination of housing, personal support and health care while promoting autonomy, privacy and independence [12]. A facility was deemed eligible if it had been in operation for at least 6 months, did not primarily serve clients with mental illness or developmental disabilities and housed a minimum number of DAL residents aged 65 and older (≥ 4 for small and ≥ 10 for large facilities, respectively). Of 60 DAL facilities approached, 59 agreed to participate.

**Figure 1:**
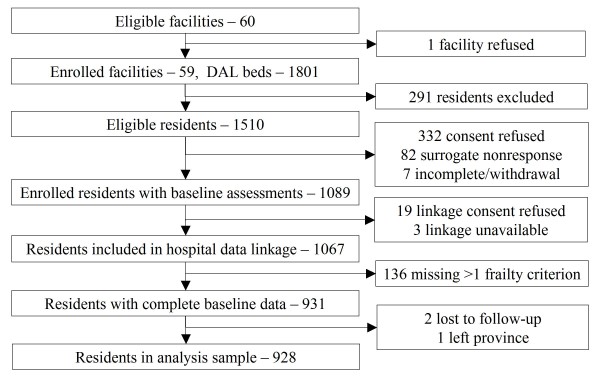
**Alberta Continuing Care Epidemiological Study (ACCES) - AL Cohort**.

All eligible DAL residents within these facilities were approached for participation. Residents were excluded if they were less than 65 years of age, recently admitted (< 21 days), or receiving palliative care (with an expected survival < 6 months and/or whose participation was otherwise deemed inappropriate by staff or family). A total of 1,089 participants were enrolled and assessed with 1,067 providing consent for linkage with administrative data (see Figure 1).

Ethics approval was obtained from the University of Calgary Conjoint Health Research Ethics Board, the University of Alberta Health Research Ethics Board and the University of Lethbridge Human Subject Research Committee. Administrative approvals from the health regions and/or facilities were also obtained.

Trained research nurses (RNs) administered the Resident Assessment Instrument for Assisted Living (inter*RAI-AL*) and frailty measures at baseline (2006-2007) and at 1-year. The inter*RAI-AL *tool is a comprehensive, standardized assessment of residents' sociodemographic characteristics, physical and cognitive status, health conditions, behavioural problems, and use of medications and services [13]. Resident data were linked with the Alberta Inpatient Discharge Abstract Database for fiscal years 2002-03 to 2008-09. This administrative database captures essentially 100% of hospital admissions in the province.

### Frailty criteria

Slow gait speed, muscle weakness (grip strength), low activity level, self-reported weight loss and exhaustion were assessed at baseline with residents categorized as "normal" or "impaired" using the methods described in Table 1. As noted, two different interpretations of these criteria were employed: one used CHS-specified cut-points (*absolute cut-points*) for determining select criteria (i.e., gait speed, grip strength, physical activity) while the second was norm-referenced and identified the poorest performers for that criterion within the AL population (*relative cut-points*). Also, as described in Table 1, our measures for physical activity, weight loss and exhaustion were slightly modified from the original CHS items [5]. For both interpretations, those classified as impaired on three or more of these criteria were defined as frail, those impaired on one or two criteria as pre-frail, and those not impaired on any as not frail.

**Table 1:** Details of measures and cut-points employed for select frailty criteria

Criterion	Measure	CHS-specified^1 ^absolute cut-points	AL population-based relative cut-points
*Slow gait*	Determined by taking the better of two timed 3-meter walks.	≥ 7 seconds^2^, men ≤ 173 cm ≥7 seconds, women ≤159 cm ≥ 6 seconds, men > 173 cm ≥ 6 seconds, women > 159 cm	*Slowest quartile*^3 ^of walk times: > 9 seconds, men > 10 seconds, women
*Muscle weakness*	Average of three grip strength readings using a handheld dynamometer.^4^	BMI-specific thresholds: ≤ 29-32 kg, men ≤ 17-21 kg, women	*Lowest quartile*‡ of grip strength readings: < 15 kg, men < 7 kg, women
*Low physical activity*	Reported minutes over two weeks per activity type - from the inter*RAI-AL "*Exercise or Leisure Activities" ^5^	Activities were mapped to Minnesota Leisure Time Activity Questionnaire.^6 ^Kcals per week calculated based on the intensity codes: < 383 Kcals/week, men < 270 Kcals/week, women	< 140 minutes/two weeks (< 10 minutes/day on average^7^)
*Unintentional weight loss*	Answer to question: "In the past year have you lost more than 10 pounds unintentionally" ^8^	Response of Yes	Response of Yes
*Exhaustion*	Answers to 3 questions: "In the past month, on average, have you been: 1) Feeling unusually tired during the day?; 2) Feeling unusually weak?; and/or, 3) Feeling an unusually low energy level?"^9^	Response of Yes to any of the 3 questions	Response of Yes to any of the 3 questions

CHS = Cardiovascular Health Study, AL = assisted living, cm = centimeters, BMI = body mass index, kg = kilograms, kcals = kilocalories.

^1 ^as detailed in Fried et al., 2001 [5]

^2 ^sex and height-specific thresholds.

^3 ^a standard cut-point [32].

^4 ^JAMAR^®^, Sammons Preston Rolyan, Bolingbrook, IL.

^5 ^include: aquasize/swimming; bowling; dancing; exercise bike/treadmill; exercise program; floor curling/lawn bowling; gardening; household chores; shuffleboard/pool; Tai chi/yoga; walking/wheeling indoors & outdoors.

^6 ^as detailed in [45].

^7 ^levels above this cut-point shown to be beneficial in otherwise sedentary people [33].

^8 ^CHS also allowed actual unintentional 5% weight loss over 1-year (not assessed in ACCES).

^9 ^CHS used 2 items from the CES-D Scale [46]: "I feel that everything I do is an effort" and "I cannot get going" (those reporting feeling this way at least 3-4 days/previous week fulfilled the criterion).

### Outcome measures and baseline characteristics

Primary outcomes were one-year mortality (recorded at the 12-month follow-up based on reviews of facility discharge records, family interviews and obituaries) and hospitalizations (determined via linkage with the Alberta Inpatient Discharge Abstract database).

A baseline co-morbidity score was calculated based on the Charlson index [14] and a validated coding algorithm [15] using relevant diagnostic codes (any occurrence during 3 years prior to baseline) from the inpatient database. An additional co-morbidity measure was created from the sum of recorded diagnoses on the inter*RAI-AL *tool. Other baseline characteristics included three validated scales derived from items on the inter*RAI-AL *tool: the Cognitive Performance Scale (CPS) [16], Depression Rating Scale (DRS) [17], and Activities of Daily Living (ADL) Self-Performance Hierarchy Scale [18].

### Missing data for CHS frailty criteria and value assignment

Of the 1,067 residents consenting to data linkage, it was possible to assess all five CHS frailty criteria for 648 (60.7%). File comments by the RNs indicated that missing values usually occurred when residents refused or failed to comprehend what was requested. Over half (58%) of the cohort had a diagnosis of dementia. Consequently, additional (observed) functional and health items assessed as part of the inter*RAI-AL *tool were used to assign values in the following manner.

Gait speed was missing for 287 (26%) residents. If the RN documented that at least "limited assistance" was required with "walking between locations on the same floor indoors" and that "in the last 3 days the longest distance walked without sitting down was less than 5 meters", the resident was coded as impaired. This reduced missing gait speed values to 97 (9% of total group). Muscle weakness was missing for 105 residents (10%). Impairment was determined as present if RNs stated the resident was physically unable to perform the grip test. This reduced missing muscle weakness values to 74 (7%). Missing data on weight loss and exhaustion occurred in 116 (11%) and 149 (14%) of residents respectively. Assignment for these variables was based on yes/no responses to inter*RAI-AL *items as assessed by RNs. A positive response to the item, "Subject has had weight loss of 5% or more in last 30 days, or 10% or more in last 180 days" was used for weight loss while a positive response to the item "Due to diminished energy, is unable to finish normal day-to-day activities, or start some or any normal day-to-day activities" was used for exhaustion. This reduced missing values to two for weight loss, and zero for exhaustion.

Of the enrolled cohort of 1,089, 22 residents refused consent for administrative data linkage (or no linkage was available); 136 had one or more missing frailty value(s) (*after value assignment*); two were lost to follow-up, and one moved out of province after baseline (total of 161 excluded). The final analysis sample was 928 - see Figure 1.

### Analysis

Generalized linear models with a binomial distribution and log link were used to estimate risk ratios for analyses. Multivariable models adjusting for age, sex, and co-morbidity were examined to assess the prognostic significance of each criterion. The risks of one-year mortality and hospitalization for those categorized as frail or pre-frail by CHS-specified absolute cut-points and AL relative cut-points (compared with non-frail residents) were assessed in multivariable models. The models considered potential effect modification and confounding by age, sex and co-morbidity. Predictive accuracy was assessed by comparing the area under the receiver operating characteristic curve (AUC) of a baseline model with only age and sex with that of models with age/sex/co-morbidity, age/sex/frailty, and age/sex/frailty/co-morbidity respectively. To facilitate model comparisons, 95% confidence intervals for differences in AUC estimates (with associated p-values) were calculated as has been reported by others [19].

The level of clustering of residents within facilities was quantified by calculating the design effect as 1+(*M*-1)*r*, where *M *is the mean cluster size and *r *is the estimated intraclass correlation coefficient. The mean cluster size was 15.7 residents among the 59 facilities and the estimated intraclass correlation coefficient was 0.0012, resulting in a design effect of 1.018. As the magnitude of the design effect was small (and because adjustment for clustering did not appreciably alter our model findings), we have presented the results of our main analyses without adjustment for clustering - which permits the use of simpler statistical approaches without the loss of information. SAS Version 9.2 (SAS Institute, Inc., Cary, NC) was used for analyses.

## Results

The enrolled cohort was predominantly female (76.7%) with an average age of 84.9 years. For 364 of the 421 eligible residents not enrolled, age and sex were available and showed a similar distribution (73.1% female; mean age 84.4) to the enrolled cohort.

Most baseline characteristics of the analysis sample did not differ significantly from the enrolled cohort (Table 2), although there was a lower proportion with moderate/severe cognitive impairment (24.1% vs. 28.5%) and extensive impairment/dependency in ADL (25.3% vs. 28.3%). Residents excluded because of missing data (n = 161) showed a significantly greater proportion with slow gait, muscle weakness, low activity, cognitive & ADL impairment, and depression.

**Table 2:** Baseline characteristics for ACCES - AL cohort

	All baseline (n = 1,089)^1^	Missing data (n = 161)^2^	Complete, linked data (n = 928)
Age, mean ± SD	84.9 ± 7.3	85.0 ± 7.3	84.9 ± 7.3
Female, n (%)	835 (76.7)	134 (83.2)	701 (75.5)
Charlson co-morbidity index, mean ± SD	1.8 ± 2.0^1^	1.8 ± 2.0^2^	1.8 ± 2.0
inter*RAI-AL *co-morbidity count	4.5 ± 1.9	4.4 ± 1.9	4.5 ± 1.9
Cognitive Performance Scale (CPS)			
Intact/borderline intact (score 0-1)	437 (40.1)	38 (23.6)	399 (43.0)
Mild impairment (score 2)	342 (31.4)	36 (22.4)	306 (33.0)
Moderate impairment (score 3-4)	193 (17.7)	42 (26.1)	151 (16.3)
Severe/very severe impairment (score 5-6)	117 (10.7)	45 (27.9)	72 (7.8)
Depressive symptoms (DRS 3+)	209 (19.2)	50 (31.1)	159 (17.1)
Activities of Daily Living (ADL) Scale			
Independent (score = 0)	458 (42.1)	31 (19.3)	427 (46.0)
Supervision required (score = 1)	189 (17.4)	29 (18.0)	160 (17.2)
Limited impairment (score = 2)	134 (12.3)	28 (17.4)	106 (11.4)
Extensive assistance required (score = 3-4)	247 (22.7)	52 (32.3)	195 (21.0)
Dependent (score = 5-6)	61 (5.6)	21 (13.0)	40 (4.3)

Frailty criteria^3^, n (%)			
• Slow gait - absolute	695 (70.1)^1^	56 (87.5)^2^	639 (68.9)
• Slow gait - relative	346 (34.9)^1^	40 (62.5)^2^	306 (33.0)
• Muscle weakness - absolute	902 (88.9)^1^	81 (93.1)^2^	821 (88.5)
• Muscle weakness - relative	262 (25.9)^1^	33 (38.0)^2^	229 (24.7)
• Low physical activity - absolute	415 (38.1)	81 (50.3)	334 (36.0)
• Low physical activity - relative	403 (37.0)	74 (46.0)	329 (35.5)
• Unintentional weight loss	164 (15.1)^1^	27 (17.0)	137 (14.8)
• Exhaustion	391 (36.0)	56 (35.0)	335 (36.1)

**Frailty: CHS-specified *absolute cut-points***			
Not frail, score = 0			32 (3.4)
Pre-frail			
score = 1			157 (16.9)
score = 2			294 (31.7)
Frail, score = 3+			445 (48.0)

**Frailty: AL population-based *relative cut-points***			
Not frail, score = 0			238 (25.6)
Pre-frail			
score = 1			294 (31.7)
score = 2			218 (23.5)
Frail, score = 3+			178 (19.2)

ACCES = Alberta Continuing Care Epidemiological studies, AL = assisted living, SD = standard deviation, CHS = Cardiovascular Health Study.

^1^Denominators accounting for missing values: Charlson n = 1,067; slow gait n = 992; muscle weakness n = 1,015;

weight loss n = 1,087.

^2^Excluded observations due to missing frailty criteria, not linking to hospital data, loss to follow-up. Denominators

accounting for missing values: Charlson n = 139; slow gait n = 64; muscle weakness = 87; weight loss n = 159.

^3^As defined in Table 1.

The CHS absolute cut-point for gait speed categorized 69% of AL residents as impaired (Table 2). Although the relative cut-point was determined based on the lowest quartile for performance, 33% were categorized as impaired after assigning those with missing data. The absolute cut-point for grip strength categorized 89% of residents as impaired compared to 25% using the relative cut-point. Both definitions of total physical activity (kcals compared to minutes per day) categorized a similar proportion (36%) as having low activity. Only 5% of residents categorized as impaired in total physical activity using the CHS interpretation were not categorized as impaired by the relative interpretation (and vice versa).

The proportion of residents categorized as frail was 48% (445) using the CHS absolute cut-points and 19.2% (178) using the relative cut-points. The proportions defined as not frail (robust) or pre-frail were 3.4% (32) and 48.6% (451) using absolute cut-points and 25.6% (238) and 55.2% (512) with relative cut-points, respectively. During follow-up, there were 142 deaths (one-year mortality rate of 15.3%), and 375 residents had at least one hospitalization (one-year hospitalization rate of 40.4%). Of the individual frailty criteria, the best predictors of death in a model adjusting for age, sex, co-morbidity index and other frailty criteria were the relative definition of slow gait (risk ratio [RR] 1.36, 95% confidence interval (CI) 1.01-1.83), either definition of low physical activity (RR 1.60, 95% CI 1.19-2.16 for absolute and RR 1.50, 95% CI 1.11-2.03 for relative cut-points), and exhaustion (RR 1.61, 95% CI 1.20-2.15) (see Table 3). The best predictors of hospitalization in a fully-adjusted model were the absolute definition of slow gait (RR 1.23, 95% CI 1.02-1.49) and either definition of low physical activity (RR 1.18, 95% CI 1.01-1.38 for absolute and RR 1.24, 95% CI 1.04-1.47 for relative cut-points).

**Table 3:** One-year death and hospitalization outcomes for selected frailty criteria, Risk Ratios (95%Confidence Intervals), n = 928

	Death		Hospitalization	
	
Frailty Criteria	Absolute Definition	Relative Definition	Absolute Definition	Relative Definition
Slow gait - absolute				
Model 1	1.40 (0.98-1.99)		1.34 (1.11-1.62)	
Model 2	1.34 (0.93-1.91)		1.29 (1.07-1.56)	
Model 3	1.31 (0.92-1.86)		1.23 (1.02-1.49)	
Slow gait - relative				
Model 1		1.57 (1.17-2.10)		1.20 (1.03-1.41)
Model 2		1.53 (1.14-2.05)		1.17 (1.00-1.37)
Model 3		1.36 (1.01-1.83)		1.07 (0.90-1.28)
Weakness - absolute				
Model 1	0.93 (0.56-1.54)		1.04 (0.80-1.34)	
Model 2	0.88 (0.53-1.47)		1.00 (0.80-1.29)	
Model 3	0.73 (0.44-1.22)		0.93 (0.72-1.19)	
Weakness - relative				
Model 1		1.15 (0.84-1.58)		1.18 (0.99-1.39)
Model 2		1.11 (0.81-1.53)		1.15 (0.97-1.36)
Model 3		0.96 (0.70-1.32)		1.11 (0.93-1.33)
Low activity - absolute				
Model 1	1.70 (1.26-2.29)		1.27 (1.09-1.49)	
Model 2	1.66 (1.23-2.24)		1.24 (1.07-1.45)	
Model 3	1.60 (1.19-2.16)		1.18 (1.01-1.38)	
Low activity - relative				
Model 1		1.68 (1.25-2.27)		1.29 (1.11-1.51)
Model 2		1.69 (1.25-2.26)		1.31 (1.12-1.52)
Model 3		1.50 (1.11-2.03)		1.24 (1.04-1.47)
Weight loss				
Model 1	1.29 (0.89-1.87)	same as at left	1.31 (1.09-1.58)	same as at left
Model 2	1.18 (0.81-1.71)	same as at left	1.18 (1.00-1.40)	same as at left
Model 3	1.05 (0.73-1.52)	1.01 (0.70-1.46)	1.19 (0.99-1.42)	1.16 (0.95-1.42)
Exhaustion				
Model 1	1.73 (1.30-2.32)	same as at left	1.22 (1.05-1.43)	same as at left
Model 2	1.65 (1.23-2.22)	same as at left	1.16 (0.99-1.34)	same as at left
Model 3	1.61 (1.20-2.15)	1.58 (1.17-2.12)	1.13 (0.97-1.31)	1.11 (0.94-1.31)

Model 1 adjusted for age, sex; Model 2 adjusted for age, sex, Charlson index; Model 3 adjusted for age, sex, Charlson index, other four frailty criteria.

When derived from absolute cut-points, those categorized as frail were more likely to die (RR 1.75, 95% CI 1.08-2.83) and to be hospitalized (RR 1.54, 95% CI 1.20-1.96) within one year after adjusting for age, sex and co-morbidity (see Table 4). Pre-frail residents defined by absolute cut-points showed no increased risk for mortality or hospitalization within one-year. When derived from relative cut-points, the association between frailty and both mortality and hospitalization was apparently modified by sex (p = 0.09 and p = 0.03 for tests of interaction for mortality and hospitalization, respectively). Specifically, frail (but not pre-frail) women were more likely to die (adjusted RR 1.58, 95% CI 1.02-2.44) and be hospitalized (adjusted RR 1.53, 95% CI 1.25-1.87) within one year. Among males, those classified as frail or pre-frail using relative cut-points showed an increased mortality risk (adjusted RR 3.21, 95% CI 1.71-6.00 and adjusted RR 2.61, 95% CI 1.40-4.85, respectively). An increased hospitalization risk was evident for pre-frail men only (adjusted RR 1.58, 95% CI 1.15-2.17).

**Table 4:** One-year death and hospitalization: Absolute and relative frailty definitions, Risk Ratios (95%Confidence Intervals)

Death	Base model	Model 2	Model 3	Model 4	Model 5	Model 6
Female sex	0.55 (0.40-0.74)	0.58 (0.43-0.79)	0.56 (0.42-0.75)	0.59 (0.44-0.80)	0.54 (0.40-0.73)	0.57 (0.42-0.78)
Age (per year)	1.06 (1.04-1.09)	1.07 (1.04-1.09)	1.06 (1.03-1.08)	1.06 (1.04-1.08)	1.06 (1.03-1.08)	1.06 (1.04-1.09)
Co-morbidity (per index unit increase)	-	1.10 (1.03-1.17)	-	1.07 (1.00-1.14)	-	1.08 (1.02-1.15)
Frail^1 ^- absolute	-	-	1.89 (1.18-3.04)	1.75 (1.08-2.83)	-	-
Pre-frail^2 ^- absolute	-	-	0.92 (0.53-1.60)	0.89 (0.51-1.55)	-	-
Frail^1 ^- relative (females)	-	-	-	-	1.62 (1.05-2.51)	1.58 (1.02-2.44)
Pre-frail^2 ^- relative (females)	-	-	-	-	1.24 (0.77-1.98)	1.21 (0.76-1.95)
Frail^1 ^- relative (males)	-	-	-	-	3.47 (1.85-6.50)	3.21 (1.71-6.00)
Pre-frail^2 ^- relative (males)	-	-	-	-	2.80 (1.51-5.20)	2.61 (1.40-4.85)

**Area Under Curve**	**0.648**	**0.669^3^**	**0.689**	**0.700^3^**	**0.689**	**0.702^3^**

**Hospitalization**						

Female sex	0.93 (0.78-1.11)	1.01 (0.84-1.21)	0.94 (0.79-1.11)	1.01 (0.84-1.20)	1.01 (0.77-1.33)	1.05 (0.80-1.38)
Age (per year)	1.01 (0.99-1.02)	1.01 (1.00-1.02)	1.00 (0.99-1.01)	1.00 (0.99-1.01)	1.00 (0.99-1.01)	1.00 (0.99-1.01)
Co-morbidity (per index unit increase)	-	1.09 (1.05-1.12)	-	1.07 (1.04-1.10)	-	1.07 (1.04-1.10)
Frail^1 ^- absolute	-	-	1.65 (1.30-2.10)	1.54 (1.20-1.96)	-	-
Pre-frail^2 ^- absolute	-	-	1.14 (0.87-1.50)	1.12 (0.86-1.47)	-	-
Frail^1 ^- relative (females)	-	-	-	-	1.59 (1.29-1.95)	1.53 (1.25-1.87)
Pre-frail^2 ^- relative (females)	-	-	-	-	1.17 (0.93-1.47)	1.16 (0.92-1.46)
Frail^1 ^- relative (males)	-	-	-	-	1.22 (0.79-1.88)	1.18 (0.77-1.81)
Pre-frail^2 ^- relative (males)	-	-	-	-	1.80 (1.31-2.47)	1.58 (1.15-2.17)

**Area Under Curve**	**0.520**	**0.600^4^**	**0.597**	**0.631^4^**	**0.593**	**0.633^4^**

^1^Frail defined as having 3+ of the following as defined in Table 1: slow gait, muscle weakness, low activity, weight loss & exhaustion. Reference group consists of those with 0 criteria (non-frail).

^2^Pre-frail defined as having 1 or 2 of the above frailty criteria. Reference group consists of those with 0 criteria (non-frail).

Note:

#Deaths/Sample: total = 142/928; Absolute cut-points (Frail = 95/445, Pre-frail = 44/451, Non-frail = 3/32); Relative cut-points (Females: Frail = 27/137, Pre-frail = 55/384, Non-frail = 11/180; Males: Frail = 16/41, Pre-Frail = 26/128, Non-frail = 7/58).

#1+ Hospitalizations/Sample: total = 375/928; Absolute cut-points (Frail = 219/445, Pre-frail = 144/451, Non-frail = 12/32); Relative cut-points (Females: Frail = 75/137, Pre-frail = 148/384, Non-frail = 56/180; Males: Frail = 17/41, Pre-Frail = 60/128, Non-frail = 19/58).

^3 ^AUC estimate difference with 95% confidence interval (CI) and p-value comparing:

(i) Model 2 and Model 4 is 0.031 (95% CI .002 to .060), p-value 0.03.

(ii) Model 2 and Model 6 is 0.033 (95% CI .003 to .063), p-value 0.03.

(iii) Model 4 and Model 6 is 0.002 (95% CI -.024 to .027), p-value 0.90.

^4 ^AUC estimate difference with 95% confidence interval (CI) and p-value comparing:

(i) Model 2 and Model 4 is 0.031 (95% CI .002 to .059), p-value 0.03.

(ii) Model 2 and Model 6 is 0.033 (95% CI .005 to .060), p-value 0.02.

(iii) Model 4 and Model 6 is 0.002 (95% CI -.021 to .025), p-value 0.87.

The model for mortality with only age and sex as independent variables showed an area under the curve (AUC) of 0.648 (see Table 4). Adding co-morbidity increased it to 0.669. Including frailty based on absolute cut-points instead of co-morbidity (Model 3) gave an AUC of 0.689 with a similar value if relative cut-points were used (Model 5). A model with age, sex, co-morbidity and frailty had an AUC of 0.700 and 0.702 using the absolute (Model 4) and relative (Model 6) cut-points respectively.

For hospitalization, a base model with age and sex alone performed poorly (AUC = 0.520). The addition of co-morbidity resulted in an AUC of 0.600 - slightly higher than the models with frailty (AUC = 0.597 absolute, 0.593 relative). Including both co-morbidity and frailty led to an AUC of 0.631 and 0.633 for absolute and relative frailty cut-points respectively.

For mortality and hospitalization, the fully adjusted models with the absolute (Model 4) or relative (Model 6) frailty measures did not differ significantly from each other but both were associated with significantly higher AUCs (predictive accuracy) compared with Model 2 (with age, sex and co-morbidity only).

The above findings were essentially unchanged in models incorporating a co-morbidity count derived from diagnoses recorded on the inter*RAI-AL *tool.

## Discussion

The AL population is often described as 'frail', yet surprisingly it represents a care setting little studied in frailty research. The CHS frailty criteria have been widely examined in community-dwelling older populations. They have been investigated within diverse settings and cultural groups [20-22] and among patients suffering from a wide range of diseases (cardiovascular, renal, cancer, HIV infection [23-26]). The predictive accuracy of the CHS frailty measure has also been investigated in various cohorts of older adults and with regard to diverse health outcomes [27-29]. Our study is the first to examine the CHS criteria among a large representative sample of (designated) AL residents. Our findings raise questions about their feasibility and predictive accuracy for this population. Complete CHS criteria could not be assessed for nearly 40% of the 1,067 residents in the study sample - indicating the challenges in applying these measures in the AL setting. Although it was possible to reduce the number of missing observations with supplemental (observed) data items, the prognostic utility of the criteria remained limited. After considering age, sex and co-morbidity, CHS-defined frailty using either approach (absolute or relative) was associated with a marginally (although statistically significant) increased risk of death and hospitalization over one year. The relative cut-points identified a smaller at-risk group than the use of absolute cut-points but overall did not perform better in predicting death or hospitalization. Of note, the AUCs observed for the best frailty models (0.702 for mortality and 0.633 for hospitalization at one year) in our AL sample, although comparable with those observed in studies of community-dwelling older adults [19,30,31], illustrate models which offer only modest discriminatory power.

Although both frailty measures produced adjusted models with comparable predictive accuracy, the relative measure illustrated potentially interesting sex-differences with regard to the differential detection of risk across levels of vulnerability among AL residents. Further, the use of this relative frailty measure demonstrated the importance of considering possible competing risks when examining the prognostic significance of selected frailty measures for particular outcomes of interest (e.g., hospitalization or institutionalization). Specifically, the failure to observe a significantly increased hospitalization risk for frail men in the present study was a consequence of their particularly high mortality risk within the one year follow-up.

Of the five CHS criteria, slow gait and low physical activity most consistently predicted death and hospitalization. There is growing evidence of the significance of slow gait speed as an independent predictor of health outcomes, in particular all-cause mortality, among older community-dwelling adults [19,32]. Consistent with this research [32], residents in the lowest quartile of gait speed performance (compared with those above this cut-point) had a significantly increased risk for all-cause mortality at one year (RR 1.53, 95%CI 1.14-2.05) after adjusting for age, sex and co-morbidity. Our somewhat weaker estimate of this association relative to others [32] may reflect our shorter follow-up time, choice of comparison (reference) group and more functionally impaired sample. Both the CHS measure and our relatively simple measure of low physical activity produced comparable findings. This is of interest since our measure (physical activity < 10 minutes/day) would be easy to assess among AL residents and potentially amenable to simple interventions designed to improve activity levels and outcomes in this setting [33]. Exhaustion was significantly associated with mortality but only weakly associated with hospitalization after adjusting for age, sex and co-morbidity. The performance of the weakness and weight loss measures in adjusted models was less compelling, particularly for mortality. Interestingly, neither co-morbidity score (Charlson or that derived from recorded diagnoses on the inter*RAI-AL *tool) greatly improved the performance of our mortality models in terms of increasing the AUCs; whereas the addition of co-morbidity did result in models with improved predictive accuracy for hospitalization.

The feasibility and predictive accuracy concerns raised by our findings likely reflect, in part, the inherent differences between our AL population and the community-based population used to derive the CHS criteria and frailty 'phenotype' [5]. Notable differences include a significantly greater burden of dementia (58%), depression (34%) and other co-morbid conditions and higher average age (84.9 vs. 72.8) among AL residents compared with CHS participants [34]. Also common in the AL setting were disability-associated conditions (e.g., arthritis 54%, osteoporosis 32%) which may interfere with some physical measures (e.g., grip strength). As also noted by others [19], mobility measures (e.g., gait speed) may be less informative among older adults who are already dependent in basic ADLs (approximately 25.3% of our AL sample). Our one-year mortality rate (15.3%) was nearly double that observed for community-dwelling Albertans (8.2%) with a comparable age/sex distribution [35]. Such findings suggest that different approaches (and possibly measures) may be required in the operationalization of frailty among AL residents [20,36-39].

A single standard frailty measure used in all settings would allow comparisons of vulnerable populations across care settings and the tracking of individuals as they transition from one setting to the next. Such a single measure, though, may be problematic depending on its composition and definition. Some measures may not be feasible or may have limited discriminatory ability (e.g., due to floor effects) in more disabled populations. For gait speed, different cut-points have been suggested for less and more disabled populations [40]. This may allow one to better predict which members of a relatively more functionally impaired population are at greater risk for further decline or other adverse outcomes. The more vulnerable sub-groups within community, AL and nursing home settings are likely to be at different positions along the continuum of frailty. Anticipating that AL residents would be farther along this continuum than the original CHS population, we chose to compare an absolute with a relative operational definition of frailty. Whether frailty as conceptualized by the CHS investigators [27] is a useful construct in the AL setting and the value-added of various approaches to identifying frailty in AL residents, are important questions that should be pursued. It is plausible that the domains captured by any one measure (e.g., physical, cognitive, psychosocial) of vulnerability may differ in their relative importance in terms of predicting key outcomes of interest across different care settings [4]. Subsequent research should include comparative analyses of the utility and prognostic significance of other existing frailty measures (e.g., the Frailty Index [41], the Clinical Frailty Scale [42], the Frail Scale [43]) and/or types of measures (e.g., continuous vs. categorical) among AL residents and similar populations. Compared with the physical characteristics captured by the CHS criteria, other approaches may be more inclusive of domains (e.g., cognitive, functional and psychosocial) with specific prognostic relevance to older vulnerable adults in various care settings [3,4,9,20].

Some limitations of our study warrant consideration. Over four hundred eligible residents did not enroll in the study. Although the age and sex distribution of this sample was comparable to our enrolled cohort, this may raise concerns regarding the generalizability of our findings to the larger AL population. We restricted eligibility to residents of publicly-subsidized (or designated) AL facilities in Alberta. Our findings may not apply to residents in private AL or other AL-type facilities across Canada. Hospitalizations were captured using provincial data and may have missed the rare event that occurred outside Alberta. Our assessments for low physical activity, unintentional weight loss and exhaustion varied slightly from the specific measures used in the CHS [5]. While we could not directly compare our modifications with the original CHS measures, we believe our approach still captured these domains in a meaningful way. This limitation is not unique to our study as even Fried and her colleagues had to modify the approach used in the CHS in their analyses of frailty in the Women's Health and Aging Studies [27]. Finally, we limited our exploration of predictive accuracy to two relevant outcomes (all-cause mortality and hospitalization during one year) and further consideration of the prognostic utility of select frailty criteria to other outcomes (e.g., falls, functional decline) over longer follow-up is warranted. In regard to this latter point, we wish to re-emphasize that our primary aim was not to develop and test a comprehensive prediction model for these outcomes in our AL population. Rather, our goal was to examine the utility and prognostic significance of two different interpretations of a currently well-established frailty measure - elements of which may provide simple and practical screening tools for clinical purposes [19,32].

## Conclusions

This study represents the first attempt to examine the utility and prognostic accuracy of frailty among older AL residents. The rapid expansion of AL across North America and the significant heterogeneity in functional, psychosocial and health needs of residents in this care setting makes it an important target for frailty research. Our findings suggest that the CHS definition of frailty, based on physical performance measures and self-reported data, may have limited practical and prognostic value among residents of AL facilities. At the same time, our results highlight the need for further research on frailty in the AL setting including consideration of cognitive and social determinants [20,39,44] of health outcomes in this vulnerable population.

## Competing interests

The authors declare that they have no competing interests.

## Authors' contributions

EF was responsible for the main analysis and interpretation of the data and drafted the initial manuscript. CM and LS were responsible for the conception and design of the study, directed the acquisition of data and made substantial contributions to the analysis and interpretation of data. DH, SP and HS provided substantial input regarding clinical content and analysis/interpretation of the data. ME made substantial contributions to the study design and analysis of data. All authors were involved in revising the manuscript critically for important intellectual content and have given final approval of the version to be published.

## Pre-publication history

The pre-publication history for this paper can be accessed here:

http://www.biomedcentral.com/1471-2318/11/23/prepub
